# Discovery of Potent Degraders of the Dengue Virus Envelope Protein

**DOI:** 10.1002/advs.202405829

**Published:** 2024-08-15

**Authors:** Zhengnian Li, Han‐Yuan Liu, Zhixiang He, Antara Chakravarty, Ryan P. Golden, Zixuan Jiang, Inchul You, Hong Yue, Katherine A. Donovan, Guangyan Du, Jianwei Che, Jason Tse, Isaac Che, Wenchao Lu, Eric S. Fischer, Tinghu Zhang, Nathanael S. Gray, Priscilla L. Yang

**Affiliations:** ^1^ Department of Chemical and Systems Biology Chem‐H and Stanford Cancer Institute Stanford Medicine Stanford University 290 Jane Stanford Way Stanford CA 94305 USA; ^2^ Department of Microbiology and Immunology Stanford University School of Medicine 279 Campus Drive Palo Alto CA 94305 USA; ^3^ Department of Cancer Biology Dana‐Farber Cancer Institute 450 Brookline Avenue Boston 02215 USA; ^4^ Department of Biological Chemistry and Molecular Pharmacology Harvard Medical School 240 Longwood Avenue Boston 02115 USA

**Keywords:** antivirals, dengue, envelope protein, infection, protein degradation

## Abstract

Targeted protein degradation has been widely adopted as a new approach to eliminate both established and previously recalcitrant therapeutic targets. Here, it is reported that the development of small molecule degraders of the envelope (E) protein of dengue virus. Two classes of bivalent E‐degraders are developed by linking two previously reported E‐binding small molecules, GNF‐2, and CVM‐2‐12‐2, to a glutarimide‐based recruiter of the CRL4^CRBN^ ligase to effect proteosome‐mediated degradation of the E protein. ZXH‐2‐107 (based on GNF‐2) is an E‐degrader with ABL inhibitory activity while ZXH‐8‐004 (based on CVM‐2‐12‐2) is a selective and potent E‐degrader. These two compounds provide proof of concept that difficult‐to‐drug targets such as a viral envelope protein can be effectively eliminated using a bivalent degrader and provide starting points for the future development of a new class of direct‐acting antiviral drugs.

## Graphical abstract

Targeted protein degradation is a novel strategy for eliminating challenging therapeutic targets. Proteolysis Targeting Chimeras (PROTACs) targeting the dengue envelope protein are developed by linking pyrimidine‐derived E‐inhibitors to glutarimide‐derived recruiters of E3 CRL4^CRBN^ ubiquitin ligase. The study demonstrates that viral envelope proteins can be effectively targeted with bivalent degraders and that degradation of the envelope protein results in significant antiviral activity. This opens opportunities to use targeted protein degradation to develop antivirals against non‐enzymatic viral targets.

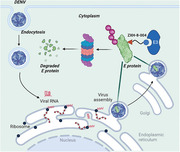

## 1 Introduction

Dengue is a mosquito‐borne tropical disease caused by dengue virus (DENV) infection. Dengue disease has affected more than 100 countries in tropical and subtropical regions, putting ≈3.6 billion people at risk.^[^ ^1^ ^]^ It was estimated that ≈390 million people are infected per year, with an estimated 96 million people experiencing severe disease.^[^ ^2^ ^]^ Dengue virus belongs to the genus Flavivirus of the Flaviviridae family, which includes several other human pathogens including Zika, yellow fever, West Nile, and Japanese encephalitis viruses (ZIKV, YFV, WNV, and JEV, respectively).^[^ ^3^ ^]^

Dengue virus strains are classified into four related serotypes of virus (DENV1‐4).^[^ ^4^ ^]^ The genome of DENV encodes three structural proteins (the envelope (E) protein, precursor membrane/ membrane (prM) / (M) protein, and capsid (C) protein), which are essential components of the viral particle, and seven non‐structural (NS) proteins (NS1, NS2A, NS2B, NS3, NS4A, NS4B, and NS5) that are responsible for replication of the viral genome and antagonism of the host immune system.^[^ ^5^ ^]^

To date, there are still no approved antiviral agents to prevent or treat DENV infection. Although the first dengue vaccine, Dengvaxia, developed by Sanofi Pasteur was licensed in December 2015, follow‐up studies demonstrated an increased risk of severe dengue and increased risk of hospitalization in seronegative individuals after Dengvaxia vaccination.^[^ ^6^ ^]^ Therefore, there remains an urgent need for new vaccines and small molecule anti‐viral agents.

The envelope (E) protein covers the surface of the DENV virion as 90 prefusion dimers atop the virus's lipid membrane. E has three distinct soluble structural domains, EDI, EDII, and EDIII.^[^ ^7^ ^]^ During viral entry, the initial attachment step is mediated by binding of EDIII on the virion to host factors on the plasma membrane surface. Following endocytosis of the virion, acidification of the endosomal compartment leads to structural changes in E that are coupled to fusion of the viral and endosomal membranes, culminating in the formation of a pore that allows the viral genome to escape to the cytosol. The DENV2 E protein was revealed to have a hydrophobic, ligand‐binding pocket located in the hinge region between EDI and EDII when a molecule of octyl‐β‐D‐glucoside (β‐OG) included in the crystallization solution co‐crystallized with the protein.^[^ ^8^ ^]^ Given the essential role of E in mediating both the attachment and fusion steps of virus entry, targeting the β‐OG pocket of the E protein with small molecules was proposed as a potential strategy for the development of a new class of antiviral agents.^[^ ^9^ ^]^ Previously, our group reported that an allosteric ABL kinase inhibitor (GNF‐2), which targets the myristate‐binding pocket in BCR‐ABL, interferes with E‐mediated membrane fusion during viral entry, with both pharmacological activities contributing to antiviral activity.^[^ ^10^ ^]^ Photoaffinity crosslinking studies and the identification of resistance mutations in the β‐OG pocket subsequently identified this site as the molecular target of these compounds.^[^ ^11^ ^]^ A subsequent medicinal chemistry effort aimed at reducing binding to BCR‐ABL resulted in the development of CVM‐2‐12‐2, a 2,4‐diamino‐substituted pyrimidine, and other analogs with increased activity against DENV2 entry compared to GNF‐2.^[^ ^10^, ^11^ ^]^ Although GNF‐2 and CVM‐2‐12‐2 provide proof‐of‐concept for antiviral activity through small molecules targeting E, the antiviral potency of these molecules^[^ ^10^, ^11^ ^]^ is currently insufficient to meet the extremely high antiviral potency required for effective antiviral drugs.

Bifunctional degraders, also known as Proteolysis Targeting Chimeras (PROTACs), have recently emerged as a promising alternative targeting modality in drug discovery.^[^ ^12^ ^]^ PROTACs are comprised of two protein‐binding ligands, one that recruits an E3 ligase and the other that binds a protein of interest (POI). Formation of the resulting ternary complex enables ubiquitination and subsequent proteasomal degradation of the POI. This strategy has been applied most frequently and successfully in oncology, with more than a dozen PROTAC drugs entering the clinical development stage by the end of 2021.^[^ ^13^ ^]^ In contrast, application of targeted protein degradation in the area of infectious disease is still an incipient field of research.^[^ ^14^ ^]^ Recently, our group developed the first small molecule anti‐viral PROTACs by using telaprevir, an established direct‐acting antiviral targeting the hepatitis C virus (HCV) NS3‐4A protease, to develop bifunctional degraders.^[^ ^15^ ^]^ Subsequently, PROTAC molecules designed to target the influenza virus neuraminidase,^[^ ^16^ ^]^ hemagglutinin,^[^ ^17^ ^]^ and PA endonuclease protein,^[^ ^18^ ^]^ and the SARS Cov‐2 main protease (M^Pro^),^[^ ^19^ ^]^ have been reported. In addition, peptide‐based PROTACs targeting the SARS CoV‐2 spike protein,^[^ ^20^ ^]^ and hepatitis B virus X protein^[^ ^21^ ^]^ have been described. With only this limited set of examples, the repertoire of viral proteins susceptible to targeted protein degradation remains largely uncharacterized. Since viruses have evolved mechanisms to ensure robust expression of their genomes, it is unclear whether highly abundant viral proteins, such as the envelope protein and other structural proteins that are needed for production of progeny virions, can be depleted sufficiently to achieve significant antiviral activity. In addition, many viral proteins may not be susceptible due to their subcellular localization.

Here, we report the development of dengue E protein‐directed PROTACs consisting of pyrimidine‐derived E‐inhibitors (GNF‐2, CVM‐2‐12‐2) conjugated to glutarimide‐derived recruiters of the E3 CRL4^CRBN^ ubiquitin ligase. A structure‐activity exploration of these two E‐binders resulted in the discovery of ZXH‐2‐107 and ZXH‐8‐004, which achieve potent on‐target degradation of the E protein. Both ZXH‐2‐107 and ZXH‐8‐004 exhibit antiviral activity against DENV2 in cell culture that is significantly higher than that of their respective parental inhibitors.

## 2 Results and Discussion

### 2.1 Development of GNF‐2‐Based E Protein Degrader

**Figure 1:**
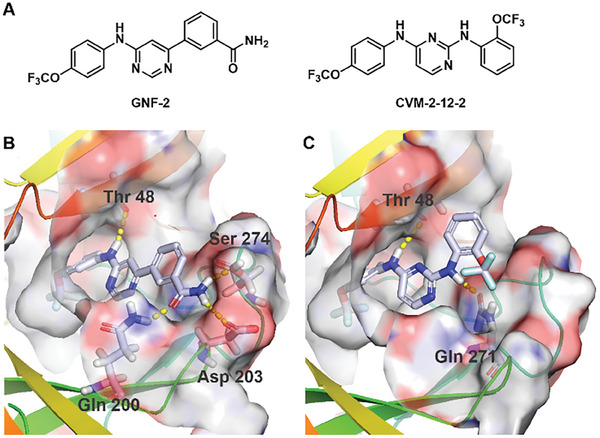
Design of PROTACs targeting dengue E. A) Chemical structures of GNF‐2 and CVM‐2‐12‐2. B) Optimal docking poses of prefusion dengue E (PDB: 1OKE) with GNF‐2 in place of β‐OG. C) Optimal docking poses of prefusion dengue E (PDB: 1OKE) with CVM‐2‐12‐2 in place of β‐OG. The E protein is shown as a ribbon cartoon and the key residues forming hydrogen bonds are represented as sticks. Docking studies were performed with Schrödinger, and models were prepared with PyMOL.

To develop a dengue E‐targeted degrader, we started with modification of GNF‐2 (**Figure** **1**A), conjugating it to a CRBN‐binding ligand derived from thalidomide. Our strategy was informed by computational docking of GNF‐2 into the hydrophobic octyl‐β‐D‐glucoside (β‐OG)‐binding pocket of E using Glide (Schrödinger suite) (Figure 1B). The model predicts that the 4‐(trifluoromethoxy) phenyl moiety is deeply embedded in the hydrophobic pocket and that the ‐NH of GNF‐2 forms a hydrogen bond with Thr48 within the pocket. In contrast, the amide group protrudes outside and is solvent‐exposed, forming three hydrogen‐bonding interactions with Gln200, Asp 203, and Ser274 near the top of the pocket. These modeling studies suggested that the amide bond is an appropriate site for the attachment of linkers.

**Figure 2:**
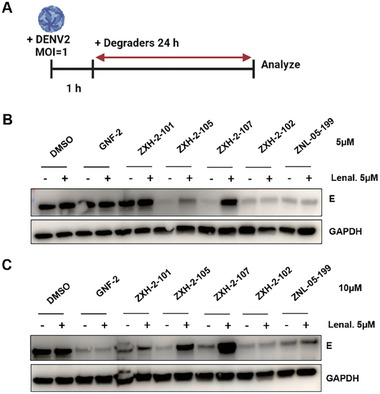
Screening of GNF‐2 based degraders. A) Schematic representation of the viral infection assay utilized to evaluate E protein degradation and antiviral activity. Created on BioRender.com with permission. B and C) Immunoblot analyses of dengue E abundance following treatment of DENV2‐infected Huh7.5 cells with GNF‐2‐based degraders at 5 and 10 µM with and without pretreatment with 5 µM lenalidomide.

Accordingly, we synthesized a series of candidate dengue E‐targeting degrader molecules by conjugating GNF‐2 through the solvent‐exposed amide position. The E3 ligase ligands of CRBN were successfully used in our previous HCV NS3/4A protease PROTACs and were examined in this work.^[^ ^15^ ^]^ Compounds were synthesized by amidation from the 3‐(pyrimidin‐4‐yl) benzoic acid, linked with either a polyethylene glycol (PEG) or an alkyl linker, and terminated at the 4‐position or 5‐position of thalidomide (**Table** **1**). To assess whether these compounds induced degradation of dengue E in cells, we examined their effects on E abundance in DENV2‐infected cells. For this, Huh 7.5 cells were infected at a multiplicity of infection (MOI) of 1 for one hour, and then incubated in the presence of the candidate E degraders. Cell lysates and culture supernatants were harvested at 24 h post‐infection to allow examination of intracellular E abundance by immunoblot and quantification of infectious progeny virus by viral plaque formation assay (PFA), respectively (**Figure** **2**A).

**Table 1:** The structures of GNF‐2 based dengue E‐degraders.

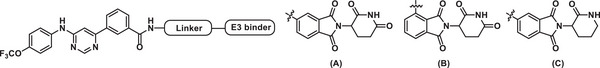		

Compound	Linker	E3 recruiter
ZXH‐2‐101	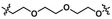	B
ZXH‐2‐107 (GNF‐2‐deg^[^ ^27^ ^]^)	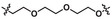	A
ZXH‐2‐102	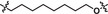	B
ZXH‐2‐105	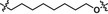	A
ZNL‐05‐199	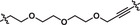	B
ZXH‐2‐107‐Neg (GNF‐2‐deg‐BUMP^[^ ^27^ ^]^)	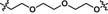	C

As shown in Figure 2B,C, several compounds caused reduction of E detected in infected cell lysates following a 24 h treatment of infected Huh 7.5 cells at 5 and 10 µM. This time point was chosen to allow correlation of degradation of E with antiviral activity, as 24 h represents the earliest time progeny virus – the product of the viral infectious cycle – is measurable under these experimental conditions. GNF‐2 also caused a strong reduction in E protein levels at 10 µM because it inhibits E‐mediated membrane fusion, thereby blocking viral entry and preventing expression of E, and additionally exerts antiviral activity through inhibition of ABL kinases.^[^ ^10^ ^]^ To evaluate whether the candidate E PROTACs exert antiviral activity through a targeted protein degradation mechanism, we performed competitive assays in which treatment of DENV‐infected Huh 7.5 cells was performed in the presence of an excess of the CRBN‐binding ligand lenalidomide. Immunoblotting showed a large reduction of E protein in lysates from cells treated with ZXH‐2‐107 alone (Figure 2B,C); however, this depletion of E was blocked in the presence of lenalidomide, indicating that the engagement of CRBN is essential for depletion of E in the presence of ZXH‐2‐107 and suggesting that this compound causes CRBN‐dependent depletion of E. Interestingly, ZXH‐2‐105 also induced strong reduction of E, however, this was only partially rescued in the presence of excess lenalidomide, which suggests that it has a different mechanism. Furthermore, ZXH‐2‐102 and ZNL‐05‐199 caused reductions in E both with and without lenalidomide pretreatment, indicating that these compounds also possess pharmacology that is independent of E3 ligase recruitment.

To characterize ZXH‐2‐107′s antiviral activity, we considered several potential antiviral mechanisms. Parental inhibitor GNF‐2 and related compound GNF‐5 are allosteric inhibitors of BCR‐ABL and other ABL‐family kinases that bind in the myristate‐pocket^[^ ^22^ ^]^ and that have been previously used to develop cIAP (cellular inhibitor of apoptosis protein)‐derived PROTACs that induce degradation of BCR‐ABL.^[^ ^23^ ^]^ As mentioned previously, parental inhibitor GNF‐2 itself has a dual mechanism of antiviral activity, binding to E on incoming virions and inhibiting E‐mediated membrane fusion during viral entry while also inhibiting a post‐entry step in viral replication through its inhibition of cellular ABL kinases.^[^ ^10^ ^]^ Therefore, ZXH‐2‐107 could exert antiviral effects through inhibition or targeted degradation of ABL‐family kinases and/or inhibition or targeted degradation of dengue E.

To determine whether ZXH‐2‐107 exerts antiviral activity through targeted degradation of ABL‐family kinases, we tested whether ZXH‐2‐107 induces ABL degradation in the Huh 7.5 cells in a concentration‐dependent manner. Immunoblot analysis revealed no reduction of ABL protein levels within 8 h of treatment with ZXH‐2‐107 even at a concentration of 10 µm (Figure S1, Supporting Information). We further examined ZXH‐2‐107 for targeted degradation of ABL in K562 cells, which are chronic myelogenous leukemia (CML)‐derived cells that are dependent upon high expression of ABL‐family kinases and thus highly sensitive to ABL depletion. We did not observe depletion of ABL in the presence of up to 10 µM ZXH‐2‐107 treatment (Figure S2, Supporting Information). These data suggest that ZXH‐2‐107 is not an ABL degrader.

To evaluate whether the inhibition of ABL kinases occurs and is a source of ZXH‐2‐107′s antiviral activity, we conducted antiproliferative assays in K562 cells over a 72‐h period. GNF‐2 exhibited an IC_50_ of 0.53 µM in these experiments, whereas ZXH‐2‐107 exhibited significantly less potent activity with an IC_50_ of 6.6 µM (Figures S3 and S4, Supporting Information), which is correlated with its decreased cellular permeability (Table S1, Supporting Information). To determine if the stability of ZXH‐2‐107 limits its activity against K562 cells, we examined its stability in cell culture medium and found that it remains stable after 72 h of incubation at 37 °C (Figure S5, Supporting Information). These experiments suggest that ZXH‐2‐107′s antiviral activity derives from targeted degradation of E and also possibly through the inhibition of ABL‐family kinases.

### 2.2 Development and Optimization of a Potent E Protein Degrader

To enable a more straightforward evaluation of the antiviral potential of targeted degradation of E in the absence of the ABL inhibition exhibited by ZXH‐2‐107, we sought to develop an independent E‐degrader series using an E inhibitor devoid of ABL‐binding activity. For this we chose the 2,4‐diamino pyrimidine scaffold CVM‐2‐12‐2. Docking of CVM‐2‐12‐2 to the β‐OG‐bound prefusion DENV2 E protein (PDB: 1OKE) suggested that CVM‐2‐12‐2 engages Thr48, with a binding pose similar to that of GNF‐2, while forming a key hydrogen‐bonding interaction with Gln271. The model predicts that the *meta*‐position of the trifluoromethoxy functionality in CVM‐2‐12‐2 extends out of the pocket and is solvent‐exposed (Figure 1C).

**Figure 3:**
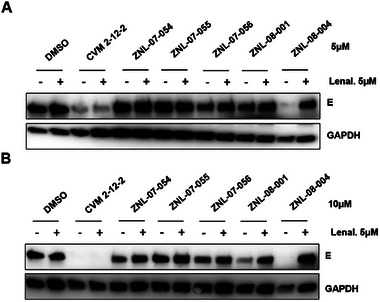
Screening of CVM‐2‐12‐2 based degraders. A) Schematic representation of the viral infection assay utilized to evaluate E protein degradation and antiviral activity. B and C) Immunoblot analyses of dengue E abundance following treatment of DENV2‐infected Huh7.5 cells with CVM‐2‐12‐2‐based degraders at 5 and 10 µM with and without pretreatment of 5 µM lenalidomide.

To make CVM‐2‐12‐2‐based degraders, we introduced a carboxylic acid at the *meta*‐position of the trifluoromethoxy group and conjugated it with linkers via amidation reaction, using thalidomide as the CRBN‐recruiting moiety. All CVM‐2‐12‐2‐based degrader molecules were evaluated with and without lenalidomide treatment in DENV2‐infected Huh 7.5 cells (MOI 1) for 24 h (**Figure** **3**A,B). ZXH‐8‐004, which contains a PEG2 linker, demonstrated the most potent depletion of dengue E after 24 h treatment at both 5 and 10 µM. Similar to GNF‐2, CVM‐2‐12‐2 caused a reduction of E at 5 and 10 µm concentrations due the antiviral activity it exerts on E‐mediated fusion during viral entry, which is notably not CRBN‐dependent. In contrast, depletion of E by candidate PROTACs is CRBN‐dependent, as demonstrated by the full rescue of E degradation in the presence of excess lenalidomide. ZXH‐08‐001, which differs from ZXH‐08‐004 only in the linkage site of the thalidomide moiety, was also observed to cause weak depletion of E at 10 µM. In our study, compounds that harbor the 4‐substituted thalidomide (ZXH‐8‐004) induced greater targeted degradation of E than those with the thalidomide at the 5‐position (ZXH‐8‐001), presumably because the location of the E3‐targeting moiety influences the conformation of the ternary complex. ZXH‐8‐004 did not induce depletion of ABL as determined by immunoblot analysis in both DENV2‐infected Huh 7.5 cells (Figure S1, Supporting Information) and K562 cells (Figure S2, Supporting Information). Consistent with the lack of interaction of CVM‐2‐12‐2 with ABL family kinases, ZXH‐8‐004 showed very weak antiproliferative activities against K562 cells (Figure S3, Supporting Information).

Having successfully identified two chemically distinct scaffolds that could be used as targeting ligands for the development of bivalent degraders of dengue E, we performed additional experiments to characterize the mechanism of action of E‐degraders ZXH‐2‐107 and ZXH‐8‐004. For this, we synthesized corresponding negative control compounds (ZXH‐2‐107‐Neg and ZXH‐8‐004‐Neg) in which the glutarimide ring on thalidomide is replaced with a δ‐lactam moiety, a modification that abrogates binding to CRBN.^[^ ^24^ ^]^ These negative control compounds enabled us to compare ZXH‐2‐107 and ZXH‐8‐004 and to distinguish between inhibition‐ and degradation‐dependent pharmacology (Tables 1 and **2**).

**Table 2:** The structures of CVM‐2‐12‐2 based dengue E‐degraders.

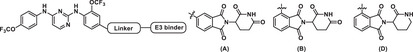		

Compound	Linker	E3 recruiter
ZNL‐07‐054	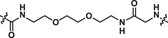	B
ZNL‐07‐055	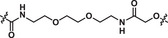	B
ZNL‐07‐056	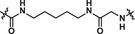	B
ZXH‐8‐001	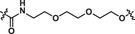	A
ZXH‐8‐004 (2‐12‐2‐de^[^ ^27^ ^]^g)	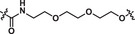	B
ZXH‐8‐004‐Neg (2‐12‐2‐deg‐BUMP^[^ ^27^ ^]^)	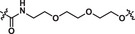	D

### 2.3 Engagement of E‐Degraders with Both CRBN and E‐Protein

**Figure 4:**
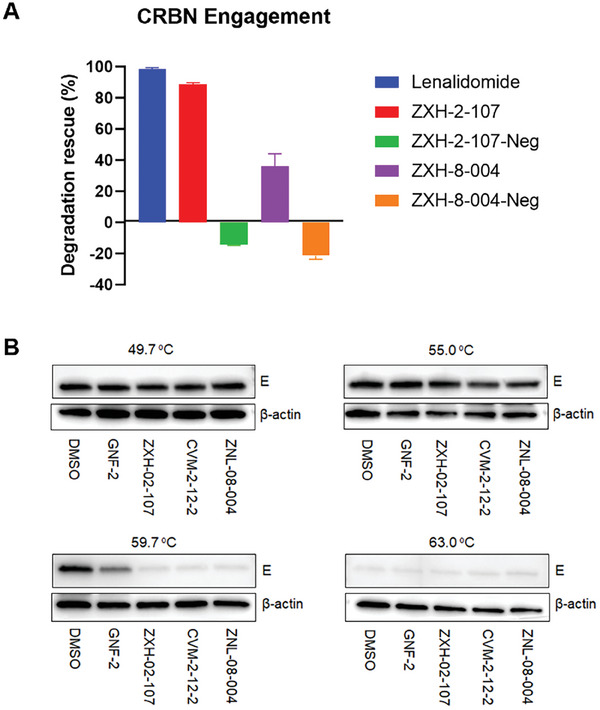
A) Cellular CRBN engagement assay of ZXH‐2‐107 and ZXH‐8‐004 at 10 µM. B) E protein CETSA for E inhibitors and degraders. The quantitative analysis of immunoblot of sample from 59.7 °C is performed with normalization of GAPDH.

To measure the CRBN engagement for the two E‐degraders and negative controls in cells, we used a previously described competitive intracellular CRBN engagement assay.^[^ ^25^ ^]^ In this assay, we measured the ability of degrader molecules to protect BRD4_BD2_ from degradation by dBET6, a pan‐BET bromodomain degrader. The assay provides a measure of the cell penetrance of degraders as well as a measure of the compounds’ ability to engage CRBN intracellularly. As expected, neither ZXH‐2‐107‐Neg nor ZXH‐8‐004‐Neg exhibited evidence of CRBN engagement (**Figure** **4**A). In contrast, both ZXH‐2‐107 and ZXH‐8‐004 engage CRBN intracellularly but not as potently as lenalidomide, most likely due to their increased molecular weights and reduced cell penetrance. Collectively, these data support that ZXH‐2‐107 and ZXH‐8‐004 are cell permeable and able to engage the CRL4^CRBN^ E3 ligase.

To assess whether ZXH‐2‐107 and ZXH‐8‐004 induced targeted degradation of dengue E by engagement of E in the cellular context, we performed cellular thermal shift assays (CETSA) in DENV2‐infected Huh 7.5 cells.^[^ ^26^ ^]^ We observed thermal‐destabilized bands in the area of 53 kDa, the anticipated size of monomeric E, in samples treated with 10 µM of GNF‐2, ZXH‐2‐107, CVM‐2‐12‐2, and ZXH‐8‐004 at 49.7, 55, 59.7, and 63 °C. Immunoblot analysis showed significant thermal destabilization of E upon treatment with both the inhibitors and the‐degraders (Figure 4B). These results demonstrate that ZXH‐2‐107 and ZXH‐8‐004 both engage E directly in cells.

### 2.4 ZXH‐8‐004 Demonstrates Potent Antiviral Activity as a CRBN‐Mediated E‐Degrader

**Figure 5:**
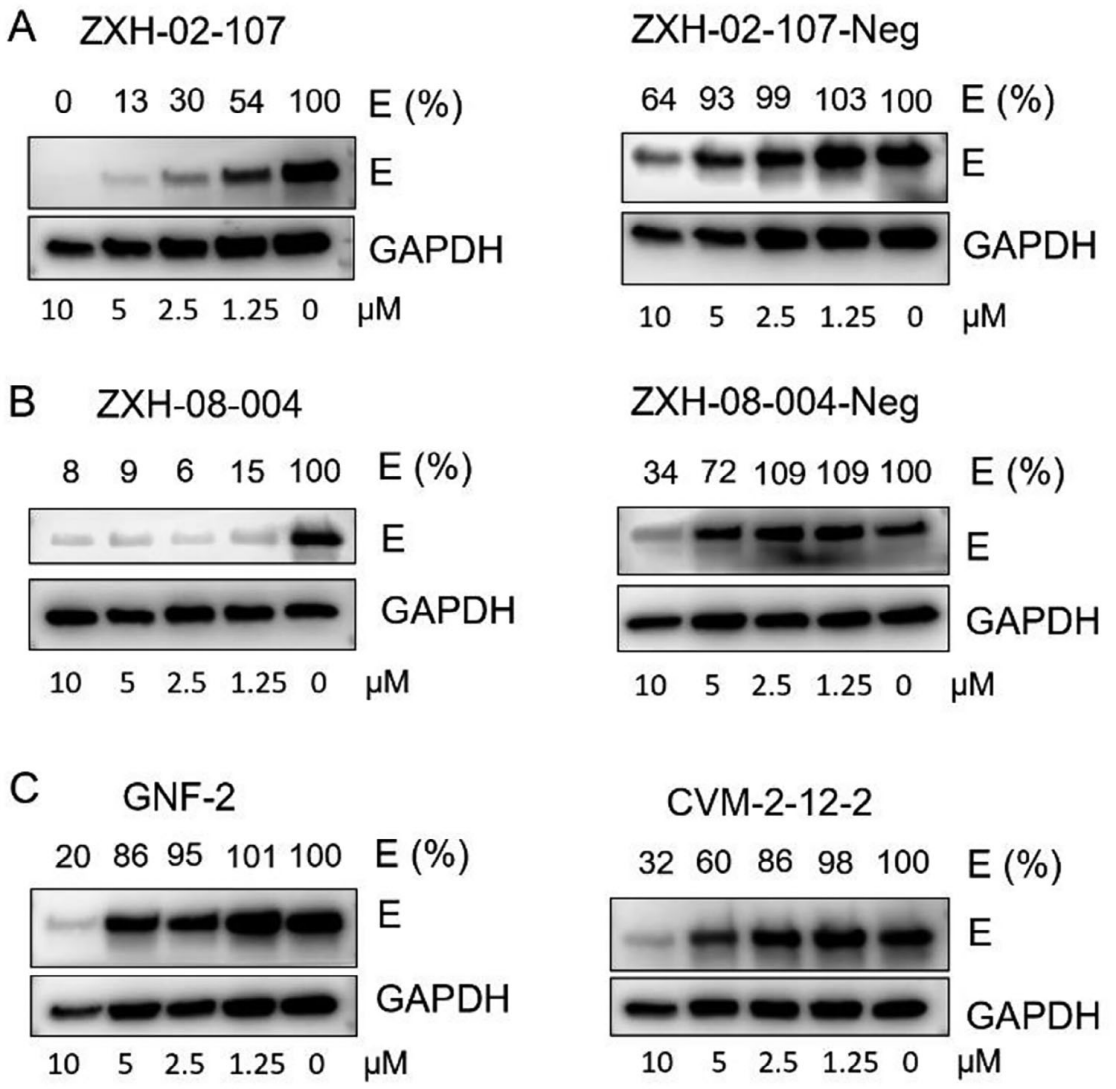
CRBN‐dependence of ZXH‐2‐107‐ and ZXH‐8‐004‐induced degradation of dengue E. A) Immunoblot analysis of E in DENV2‐infected Huh 7.5 cells treated with ZXH‐2‐107 or ZXH‐2‐107‐Neg for 24 h. B) Immunoblot analysis of E in DENV2‐infected Huh 7.5 cells treated with ZXH‐8‐004 or ZXH‐8‐004‐Neg for 24 h. C) Immunoblot analysis of E in DENV2‐infected Huh 7.5 cells treated with GNF‐2 or CVM‐2‐12‐2 for 24 h. Semi‐quantitative analysis was performed using the GAPDH signal for normalization.

We next examined the concentration‐dependent effects of ZXH‐2‐107 and ZXH‐8‐004 on E abundance in a cell culture model of DENV infection. We infected Huh7.5 cells with DENV2 (MOI 1) and treated with increasing concentrations of ZXH‐2‐107, ZXH‐2‐107‐Neg, ZXH‐8‐004, ZXH‐8‐004‐Neg, GNF‐2, and CVM‐2‐12‐2 for 24 h and then performed immunoblot analysis on cell lysates to monitor E protein abundance. As shown in **Figure** **5**A,B, ZXH‐8‐004 was more effective than ZXH‐2‐107 at concentration‐dependent depletion of E. Treatment of DENV2‐infected Huh7.5 cells with ZXH‐8‐004 reduced intracellular E starting at a 1.25 µM dose. In contrast, ZXH‐8‐004‐Neg and parental inhibitor CVM‐2‐12‐2, which cannot induce degradation of E but can inhibit E during viral entry, did not exhibit an effect on E abundance until 10 µM (Figure 5C), consistent with the interpretation that this loss of E reflects general antiviral activity and not specific depletion of E. These data indicate that low concentrations of ZXH‐8‐004 (<10 µm) yield CRBN‐dependent E degradation, while high concentrations (10 µm or above) result in depletion of E that is CRBN‐independent and may reflect inhibition of E by other mechanisms or general antiviral activity (i.e., inhibition of any step in the infectious cycle ultimately reduces the abundance of all viral proteins). In subsequent work, we have confirmed the CRBN‐ and proteasome‐dependent degrader mechanism of ZXH‐02‐107 and ZXH‐08‐004 by showing that their activity is blocked in the presence of the neddylation inhibitor MLN4924 or the proteasome inhibitor MG‐132.^[^ ^27^ ^]^

**Figure 6:**
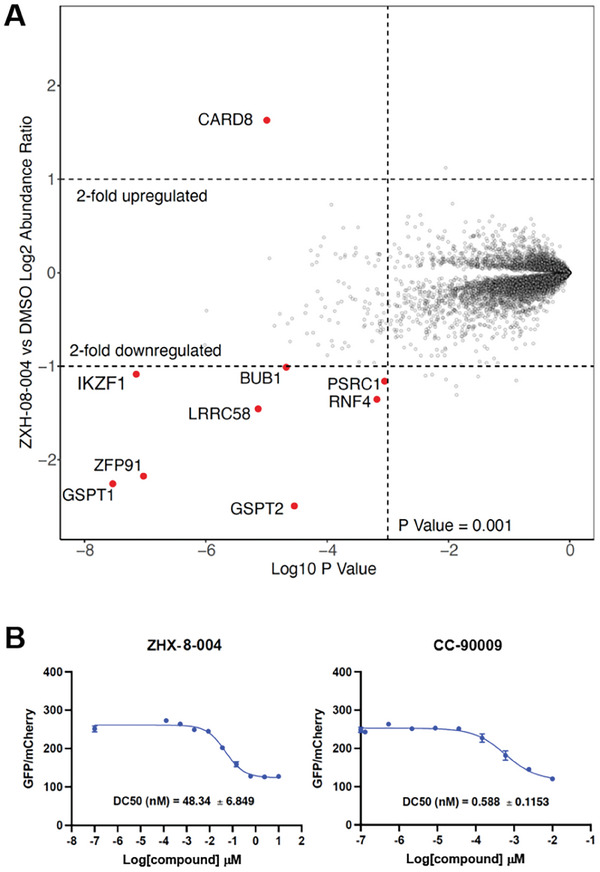
A) Proteome‐wide degradation selectivity of ZXH‐8‐004 at a dose of 3 µM in MOLT4 cells after 5 h treatment. B) Cellular GSPT1‐induced degradation of ZXH‐8‐004 and CC‐90009 assessed by reporter assay.

To assess the general selectivity of degradation on a proteome‐wide scale, we performed global quantitative mass spectrometry‐based proteomics on MOLT4 cells treated with 3 µM of ZXH‐2‐107 and ZXH‐8‐004 for 5 h, an experimental approach well‐established for proteome‐wide selectivity profiling of degraders.^[^ ^28^ ^]^ Both the mass spectrometry‐based experiments and immunoblot analysis revealed no downregulation of ABL in cells treated with ZXH‐8‐004 and ZXH‐2‐107 (**Figure** **6**A; Figure S6, Supporting Information). We did, however, observe the downregulation of zinc‐finger transcription factors such as ZFP91 and translation termination factor G1 to S phase transition 1 (GSPT1) for both compounds, which are established off‐targets of IMiD‐based degraders.^[^ ^29^ ^]^ ZXH‐2‐107 exhibited a higher number of off‐targets compared to ZXH‐8‐004, potentially due to ZXH‐2‐107′s derivation from the kinase ligand GNF‐2. Since GSPT1 is the primary off‐target^[^ ^29b^ ^]^ and previous reports suggest that GSPT1 degradation can inhibit the replication of both RNA and DNA viruses,^[^ ^30^ ^]^ we sought to address the potential contribution of GSPT1 degradation to the antiviral activity of ZXH‐8‐004. We used a stable reporter cell line expressing a GSPT1‐eGFP fusion protein fusion and mCherry reporter^[^ ^31^ ^]^ to detect depletion of GSPT1 by ZXH‐8‐004 after 24 h, corresponding to the treatment duration used to evaluate ZXH‐8‐004 in DENV2‐infected cells (Figure 3). ZXH‐8‐004 showed 80‐fold less potent degradation of GSPT1 relative to CC‐90009, a selective and potent GSPT1 degrader (Figure 6B).^[^ ^32^ ^]^ We further confirmed this by detection of endogenous GSPT1 in MOLT4 and K562 cells by Western blot following treatment (Figure S7, Supporting Information).

We then asked if GSPT1 degradation contributes to ZXH‐8‐004′s effect on E abundance and anti‐DENV activity. We first investigated the effects of the selective GSPT1 degrader CC‐90009 on the abundance of E. This was achieved by treating Huh7.5 cells infected with DENV2 (MOI 1) with escalating concentrations of CC‐90009 for 24 h, followed by examination of GSPT1 and E abundance by immunoblotting. As shown in Figure S8A (Supporting Information), E levels decrease upon treatment with CC‐90009, but this depletion is at best modest compared to the extent of GSPT1 depletion. We next evaluated the antiviral activity of CC‐90009 against infectious DENV2 in cell culture (Figure S8B, Supporting Information). CC‐90009′s potent degradation of GSPT1 was accompanied by weak antiviral activity, exhibiting ≤ 2‐fold reduction in viral titer at concentrations that reduced GSPT1 to undetectable levels. These findings show that strong depletion of GSPT1 can have a very modest effect on E abundance but suggest that ZXH‐8‐004′s effect on GSPT1 is very modest and unlikely to be a major contributor to its effects on E abundance or to its antiviral activity.

### 2.5 E‐Degraders Inhibit the Four Dengue Serotypes

**Figure 7:**
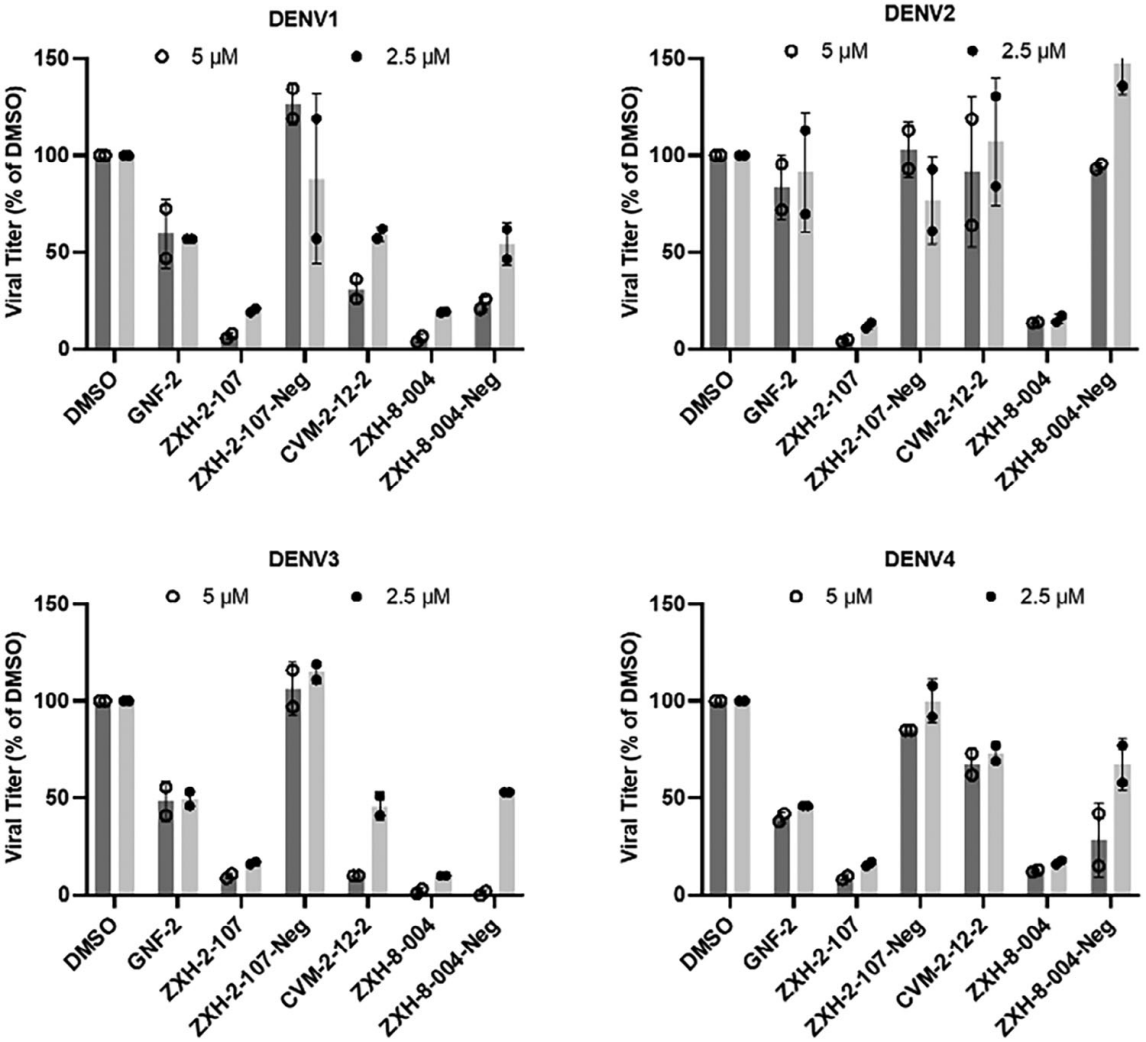
Antiviral activities of E‐degraders, negative controls, and parental inhibitors against DENV1, 2, 3, and 4 in Huh 7.5 cells at 2.5 and 5 µM. The yield of progeny virus in culture supernatants at 24 h post‐infection was quantified by viral plaque formation assay. Graphs show viral titer as % of the DMSO‐treated, DENV2‐infected controls. Viral titers are in plaque‐forming units per milliliter of culture supernatant are presented in Figure S10, Supporting Information.

Having validated ZXH‐2‐107 and ZXH‐8‐004 as on‐target E‐degraders, we next evaluated the compounds' antiviral activity against infectious DENV2 in cell culture. Both ZXH‐2‐107 and ZXH‐8‐004 exhibit significantly enhanced antiviral activity compared to their parental inhibitors, GNF‐2 and CVM‐2‐12‐2, respectively (**Figure** **7**; Figure S9, Supporting Information). We confirmed this in subsequent studies in which we measured antiviral EC_90_ values of 3.5 and 1.7 µM for ZXH‐2‐107 and ZXH‐8‐004, respectively, versus EC_90_ values of 13.1 and 13.3 µM for GNF‐2 and CVM‐2‐12‐2, respectively, against DENV2.^[^ ^27^ ^]^ Since broad‐spectrum activity across the DENV serotypes is of high interest, we additionally assessed the activity of the E PROTACs against representative strains of the other three DENV serotypes. Huh 7.5 cells were infected with each of the four DENV serotypes (DENV1‐4) at a MOI = 0.5, and the infected cells were then treated with E‐degraders at 5 and 2.5 µm concentrations. Both ZXH‐2‐107 and ZXH‐8‐004 demonstrate markedly increased antiviral efficacy across various serotypes compared to their respective parental inhibitors. This antiviral activity is CRBN‐dependent, as ZXH‐2‐107‐Neg and ZXH‐8‐004‐Neg lack the boost in potency observed for the two degrader compounds (Figure 7; Figure S10, Supporting Information).

## 3 Conclusion

Traditional direct‐acting small molecule antivirals target the functions of viral enzymes, such as polymerases and proteases. Small molecule antivirals against DENV that act against the RNA‐dependent RNA polymerase activity of NS5^[^ ^33^ ^]^ or the nonstructural protein 4b (NS4B)^[^ ^34^ ^]^ have been successfully developed although none have yet been approved. Due to the occupancy‐driven pharmacology of these compounds, they require high affinity binding to their targets to prevent viral replication and the evolution of inhibitor resistance. Since optimized degrader molecules can act catalytically, they can exert their effects at very low intracellular concentrations and may be more resilient to point mutations that reduce compound binding, a phenomenon we observed for telaprevir‐based PROTACs targeting the HCV NS3‐4A protease.^[^ ^15^ ^]^ A major challenge, however, is that we currently do not know the repertoire of viral proteins that are susceptible to targeted protein degradation. The localization of many viral processes in specialized organelles may protect viral proteins from the cellular ubiquitin‐proteasome machinery. In addition, many viruses have evolved mechanisms to ensure robust viral gene expression, raising questions about whether enough of the viral protein can be degraded to achieve significant antiviral activity. Here we explored whether previously reported small molecules that bind to the envelope (E) protein of DENV could be used as targeting moieties to generate bivalent degraders of this protein. Starting initially with GNF‐2, we were able to generate ZXH‐2‐107, which causes CRL4^CRBN^‐dependent degradation of E but retains inhibitory activity against ABL kinases. As inhibition of ABL kinase activity has been shown to inhibit DENV replication,^[^ ^10^ ^]^ we sought to generate an additional degrader that would only target E to provide a more precise pharmacological tool for assessing the antiviral effect of targeted degradation of E. To achieve this, we switched to an alternative E‐ligand, CVM‐2‐12‐2, that does not bind to ABL. Elaboration of this compound resulted in the development of ZXH‐8‐004, which effectively induces degradation of E without affecting ABL. A global proteomics experiment demonstrated that ZXH‐8‐004 has excellent selectivity for E with the only other significantly degraded proteins being the well‐known ‘neosubstrates‘ derived from the glutarimide recruiter (ZFP91 and GSPT1). We demonstrated that complete GSPT1 degradation induced by CC‐90009 has very modest anti‐DENV activity in our experimental model. Since ZHX‐8‐004 exhibits an 80‐fold less potent depletion of GSPT1 compared to CC‐90009, this indicates that GSPT1 degradation by ZHX‐8‐004 may contribute marginally or not at all to its anti‐DENV activity. Unequivocal demonstration of this should be possible with additional medicinal chemistry to differentiate the SAR for E versus GSPT1 and synthesis of E‐degraders devoid of activity against GSPT1. We also showed that degradation requires engagement with the CRL4^CRBN^ E3 ligase in competition experiments in which pretreatment with lenalidomide rescued E abundance and in experiments using negative control compounds, ZXH‐2‐107‐Neg and ZXH‐8‐004‐Neg, in which the glutarimide amide carbonyl groups have been eliminated to abrogate CRBN‐binding. These findings together demonstrate that dengue E is susceptible to targeted degradation mediated by the CRL4^CRBN^ ligase and that depletion of E from the cell by this pharmacological mechanism results in antiviral potency that exceeds that of the parental E inhibitors, which have occupancy‐driven pharmacology.

Our finding that targeted degradation of dengue E is associated with significant antiviral activity raises several important questions regarding the mode(s) of action of this activity. E, like other viral glycoproteins, has important functions in both viral entry, at the beginning of the viral life cycle, and in viral particle production, near its end. Dengue virions first attach to the plasma membrane and then are internalized to the endosome until acidification of that compartment triggers E‐mediated fusion of the viral and endosomal membranes, creating a pore through which the viral nucleocapsid can escape to the cytoplasm where the viral genome can be expressed. This process has been observed to occur on the order of minutes in live cell imaging studies,^[^ ^35^ ^]^ and it is unclear that the E3 CRL4^CRBN^ ubiquitin ligase could intercept and degrade E on the incoming virion prior to fusion. Post‐fusion, E from the incoming virion is thought to remain associated with the endosomal membrane and is presumably degraded during endosome to lysosome maturation. Post‐fusion E has no known further function for subsequent steps in the viral infectious cycle. Although targeted degradation of E could also affect the assembly of new virions, this mode of action has not been reported for classical inhibitors of dengue E or other viral envelope proteins.^[^ ^36^ ^]^ In addition, since dengue E has been thought to be co‐translationally inserted through the ER membrane to the ER lumen, this raises questions as to how and where it is accessible and susceptible to the E3 CRL4^CRBN^ ubiquitin ligase and proteasome. Last but not least, since viral structural proteins are amongst the most abundantly expressed members of a viral proteome, they might be deemed unlikely targets for antiviral targeted protein degradation. Our results, however, indicate that this class of viral proteins is indeed susceptible to targeted protein degradation and that E‐degraders can exert potent antiviral activity against all four dengue serotypes. Future work to discover what steps (entry, particle assembly) in the viral life cycle are most affected by depletion of E and the sites of interaction of E with the host ubiquitin‐proteasome system may provide valuable insights into the repertoire of viral proteins susceptible to targeted protein degradation. Relevant to our discovery, PROTAC‐based degraders of the SARS‐CoV‐2 small envelope protein have also recently been proposed,^[^ ^37^ ^]^ but this idea has, to date, not been validated experimentally. Our findings provide important proof of concept that bivalent degraders can be generated to target viral envelope proteins, providing prototype drugs as starting points for the development and optimization of antiviral degraders as a new class of direct‐acting antiviral drugs.

## Conflict of Interest

N.S.G. is a Scientific Founder, member of the SAB, and equity holder in C4 Therapeutics, Syros, Soltego (board member), Voronoi, Allorion, Lighthorse, GSK, Larkspur (board member), Shenandoah (board member) and Matchpoint. The Gray lab receives research funding from Springworks and Simcere. T.Z. is a scientific founder, equity holder, and consultant of Matchpoint, equity holder of Shenandoah. J.C. is a co‐founder and equality holder of Matchpoint Therapeutics, a scientific co‐founder of M3 Bioinformatics & Technology Inc., and a consultant and equity holder for Soltego and Allorion. E.S.F. is a founder, member of the scientific advisory board (SAB), and equity holder of Civetta Therapeutics, Lighthorse, Proximity Therapeutics, and Neomorph Inc (also board of directors), SAB member and equity holder in Avilar Therapeutics and Photys Therapeutics, and a consultant to Astellas, Sanofi, Novartis, Deerfield, Ajax and EcoR1 capital. The Fischer laboratory receives or has received research funding from Novartis, Deerfield, Ajax, Interline, and Astellas. K.A.D receives or has received consulting fees from Kronos Bio and Neomorph Inc.

## Supporting information

Supporting Information

## Data Availability

The data that support the findings of this study are available in the supplementary material of this article.
